# Supplementary material for: Urinary Potassium and Kidney Function Decline in the Population—Observational Study
Source: Nutrients. 2021 Aug 10;13(8):2747. doi: 10.3390/nu13082747 (PMC8398689; doi:10.3390/nu13082747)
Supplement: Supplementary file 1 [file nutrients-13-02747-s001.zip › nutrients-1308412-supplement.pdf]

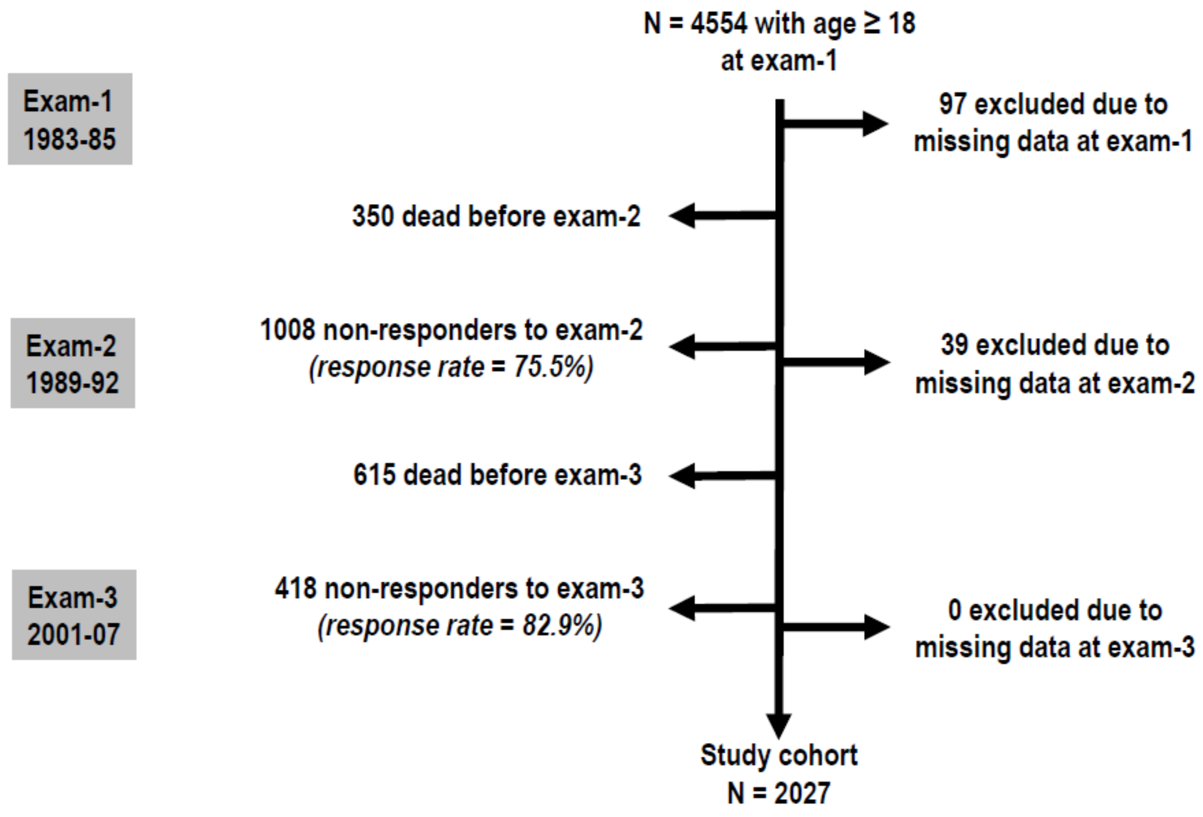

Figure S1. – Flow chart for selection of study cohort.

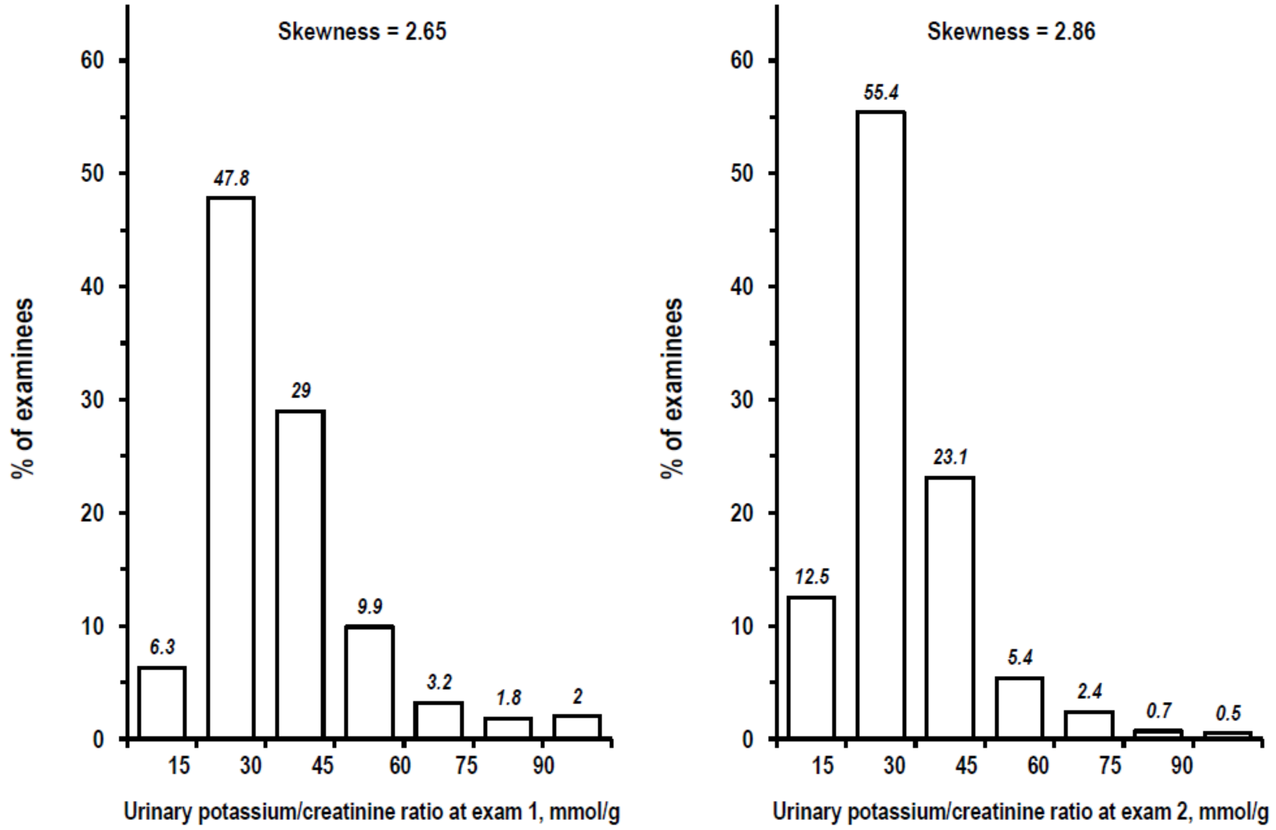

Figure S2. – Frequency distribution and skewness of uK/Cr at exam-1 (daytime spot sample) and and at exam-2 (overnight timed collection).

Table S1. - uK/Cr at exam-1 and uK/Cr at exam-2: descriptive statistics,.

differences between exams, and correlation between exams.

Non-transformed uK/Cr, mmol/g	
median (IQR) at exam-1	28.6 (21.7/38.6)
median (IQR) at exam-2	24.3 (18.7/32.9)
Z score for difference at U-test	-13.38*
Transformed uK/Cr, log mmol/g	
mean±SD at exam-1	1.47±0.20
mean±SD at exam-2	1.39±0.20
mean±SD of difference at paired t-test	0.07±0.24 *
correlation between exam-1 and exam-2	
simple coefficient	0.308*
partial coefficient§	0.247*

* P <0.001; § controlled for estimated urinary creatine and eGFR at exam-1 and exam-2.
